# Metal artifact reduction in patients with dental implants using multispectral three-dimensional data acquisition for hybrid PET/MRI

**DOI:** 10.1186/s40658-014-0102-z

**Published:** 2014-12-20

**Authors:** Jeanne M Gunzinger, Gaspar Delso, Andreas Boss, Miguel Porto, Helen Davison, Gustav K von Schulthess, Martin Huellner, Paul Stolzmann, Patrick Veit-Haibach, Irene A Burger

**Affiliations:** Department of Medical Radiology, Division of Nuclear Medicine, University Hospital Zurich, Ramistr. 100, CH-8091 Zurich, Switzerland; Global MR Applications and Workflow, GE Healthcare, CH-8048 Zurich, Switzerland; Department of Medical Radiology, Institute of Diagnostic and Interventional Radiology, University Hospital Zurich, Ramistr. 100, CH-8091 Zurich, Switzerland; Department of Medical Radiology, Institute of Neuroradiology, University Hospital Zurich, Ramistr. 100, CH-8091 Zurich, Switzerland

**Keywords:** MAVRIC, Attenuation correction, Signal voids, Image noise

## Abstract

**Background:**

Hybrid positron emission tomography/magnetic resonance imaging (PET/MRI) shows high potential for patients with oropharyngeal cancer. Dental implants can cause substantial artifacts in the oral cavity impairing diagnostic accuracy. Therefore, we evaluated new MRI sequences with multi-acquisition variable-resonance image combination (MAVRIC SL) in comparison to conventional high-bandwidth techniques and in a second step showed the effect of artifact size on MRI-based attenuation correction (AC) with a simulation study.

**Methods:**

Twenty-five patients with dental implants prospectively underwent a trimodality PET/CT/MRI examination after informed consent was obtained under the approval of the local ethics committee. A conventional 3D gradient-echo sequence (LAVA-Flex) commonly used for MRI-based AC of PET (acquisition time of 14 s), a T1w fast spin-echo sequence with high bandwidth (acquisition time of 3.2 min), as well as MAVRIC SL sequence without and with increased phase acceleration (MAVRIC, acquisition time of 6 min; MAVRIC-fast, acquisition time of 3.5 min) were applied. The absolute and relative reduction of the signal void artifact was calculated for each implant and tested for statistical significance using the Wilcoxon signed-rank test. The effect of artifact size on PET AC was simulated in one case with a large tumor in the oral cavity. The relative difference of the maximum standardized uptake value (SUV_max_) in the tumor was calculated for increasing artifact sizes centered over the second molar.

**Results:**

The absolute reduction of signal void from LAVA-Flex sequences to the T1-weighted fast spin-echo (FSE) sequences was 416 mm^2^ (range 4 to 2,010 mm^2^) to MAVRIC 481 mm^2^ (range 12 to 2,288 mm^2^) and to MAVRIC-fast 486 mm^2^ (range 39 to 2,209 mm^2^). The relative reduction in signal void was significantly improved for both MAVRIC and MAVRIC-fast compared to T1 FSE (−75%/*−*78% vs. *−*62%, *p* < 0.001 for both). The relative error for SUV_max_ was negligible for artifacts of 0.5-cm diameter (−0.1%), but substantial for artifacts of 5.2-cm diameter (−33%).

**Conclusions:**

MAVRIC-fast could become useful for artifact reduction in PET/MR for patients with dental implants. This might improve diagnostic accuracy especially for patients with tumors in the oropharynx and substantially improve accuracy of PET quantification.

## Background

In head and neck tumor staging, computed tomography (CT) and magnetic resonance imaging (MRI) play an important role in the evaluation of local tumor extension, since clinical and endoscopic examination often results in underestimation of disease, as deep infiltration of the surrounding tissues can be hard to detect [1-3]. Generally, diagnostic imaging is performed after clinical and endoscopic examination for staging and therapy planning and as a base for further follow-up examinations [4]. Functional imaging like fluorodeoxyglucose (FDG) positron emission tomography (PET) measures the metabolic activity and is superior in nodal staging compared to CT or MRI [5,6]. For accurate anatomic localization and spatial resolution, cross-sectional hybrid imaging methods like PET/CT are superior than PET alone [7,8]. For oropharyngeal cancer, T-staging could be optimized with PET/MRI compared to PET/CT, due to a higher soft tissue contrast [9,10]. This raises the interest to improve PET/MRI protocols for specific indications taking into account organ and pathology dependent adaptations [11,12]. PET/MRI has already been shown to be feasible for imaging head and neck cancer with a whole-body PET/MRI system without impairment of PET quality [13].

The two main problems for MRI of the oral cavity are patient motion and artifacts of dental alloys due to magnetic susceptibility artifacts [14]. To reduce patient motion, a short acquisition time is favorable and the patient should be well instructed and have a comfortable position [14]. The extent of artifacts from dental alloys depends on the composition, with ferromagnetic material causing strongest artifacts [15]. However, even titanium alloys generally considered ‘MRI-compatible’ may lead to significant susceptibility artifacts due to their paramagnetic properties [16]. Different MRI sequences are differently prone to those susceptibility artifacts depending on the spin excitation technique, data acquisition strategy, and receiver bandwidth [17-20]. Artifacts might appear as signal voids, hyperintense signals caused by signal pile-up due to distortion of spatial encoding, or geometric distortions [15,18,21]. An optimized MRI sequence design can reduce these artifacts significantly [14] and thereby improve diagnostic accuracy and also reduce artifacts for MR-based attenuation correction (AC), since large signal voids can lead to substantial underestimation of FDG uptake within the area of the artifact when MRI-based AC is performed [22].

Conventional strategies to optimize the image quality close to metal implants are a high bandwidth per voxel, 3D spatial encoding, a high-resolution matrix, and a multiecho spin-echo (SE) sequence or turbo/fast SE sequence [23].

The relatively new multi-acquisition variable-resonance image combination (MAVRIC) as well as the slice encoding for metal artifact correction (SEMAC) technique has shown very promising results in reducing susceptibility artifacts in arthroplasty imaging [24-27]. MAVRIC images can be used in extreme off-resonance conditions by splitting very large spectral distributions into independently imaged frequency bins with a multispectral three-dimensional technique-space composition [28]. SEMAC uses a slice selection gradient for excitation and a view-angle tilting (VAT) compensation gradient for readout [24]. MAVRIC and SEMAC showed significantly smaller artifact extent compared to fast spin-echo (FSE) imaging [24].

Given the good results of MAVRIC in arthroplasty imaging, we investigated this technique for its capability to depict the oral cavity in the presence of metallic dental implants by comparing artifacts in MRI datasets acquired with FSE, standard MAVRIC SL, and a MAVRIC-fast with an increased phase acceleration allowing a shorter repetition time (TR), resulting in notably shorter acquisition time. Furthermore, a simulation study was performed to calculate the effect of different artifact sizes on maximum standardized uptake value SUV_max_ in PET images after MRI-based AC.

## Methods

This prospective study was conducted with patients referred for FDG PET/CT who gave written informed consent for additional MRI scans during the FDG uptake time. Patients were included if they had dental implants and did not have any contraindication for MRI. Between September 2013 and January 2014, 25 patients (19 males and 6 females) were included. The study was carried out with the approval of the local ethics committee. Examinations were performed using a sequential trimodality PET/CT-MRI system consisting of a GE Healthcare Discovery 750w 3T MRI and a GE Healthcare Discovery 690 PET/CT (GE Healthcare, Milwaukee, WI, USA) [10]. A shuttle device enabling to transfer the patient from the MRI to the PET/CT without changing the patient's position was used. Standard PET/CT was acquired and axial images of the oral cavity were obtained from CT (120 kV, tube current with automated dose modulation with 60 to 440 mA/slice).

The in-phase images of a dual-echo gradient-echo pulse sequence (LAVA-Flex (GE Healthcare, Milwaukee, WI, USA) with TR 4.3 ms, echo time (TE) 1.3 ms, a matrix size of 288 × 224 with a spatial resolution of 1.7 × 2.2 × 4.0 mm; covering a field of view of 50 cm, using a bandwidth of 142.86 kHz, with an acceleration factor of 2 and a total acquisition time of 14 s) commonly used in whole-body MR imaging for AC of PET images were used as a reference [29,30]. A 2D encoded T1-weighted FSE sequence with increased bandwidth (TR 339 ms, TE 13.6 ms, slice thickness 3 mm, receiver bandwidth 142.86 kHz, acceleration factor of 1.75, acquisition time of 3.16 min) was acquired in axial orientation. Additionally, two MAVRIC sequences were applied, with 24 spectral bins of 2.25 kHz each to cover ±11 kHz (MAVRIC SL, GE Healthcare, Milwaukee, WI, USA). The standard MAVRIC SL with a phase acceleration of 2 resulted in a TR of 4,000 ms and a TE of 7.6 ms (acquisition time of 6 min). To reduce scan time, the phase acceleration was increased to 3 for MAVRIC-fast allowing a shorter TR of 3,000 ms (TE 7.6 ms), resulting in an acquisition time of 3.5 min. All three tested sequences had identical matrix sizes of 384 × 256 with an in-plane spatial resolution of 0.9 mm.

### Quantitative analysis

The signal void was quantitatively assessed for every implant using a commercially available viewing workstation (GE Advantage Windows 4.4). On the axial images of all four sequences, the largest diameter *a*_1_ and the corresponding orthogonal diameter *a*_2_ were measured by a board-certified radiologist [IAB]. The area of the artifact was calculated by assuming the shape of the artifact to be elliptical using the equation *A* = π × (*a*_1_/2) × (*a*_2_/2), with *A* meaning the area of the ellipse.

### Qualitative analysis

The qualitative image analysis was performed by two board-certified radiologists [IAB, PVH]. Both compared the four sequences independently and assessed the delineation of anatomical details of the oral cavity on a five-point scale with 1 = good depiction of anatomical structures, 2 = structures visible with slight blurring, 3 = oral cavity visible with substantial blurring, 4 = oral cavity only partially visible, and 5 = oral cavity not assessable. Furthermore, the image quality was assessed for spatial blurring and image noise on a five-point scale: 1 = no artifacts, 2 = barely visible artifacts, 3 = visible artifacts without diagnostic impairment, 4 = diagnostic impairment, and 5 = severe artifacts, non-diagnostic [27]. Hyperintense ringing artifacts around dental alloys were noted separately.

Based on the assessment of spatial blurring on LAVA-Flex sequences, two groups were generated: group 1 with low to moderate artifacts (categories 1 to 3) and group 2 with blurring artifacts impairing diagnosis (categories 4 and 5). Differences in qualitative data (anatomic distinction, blurring, or image noise) were compared for T1-FSE and MAVRIC-fast between both groups.

### MRI-based PET AC

To estimate the effect of artifact size on PET quantification if MRI sequences are used for AC, we performed a simulation analysis for one patient with a large carcinoma in the right tonsil. Therefore, artifacts of various sizes were artificially inserted into the AC atlas routinely used for the PET/MR reconstruction. The simulated artifacts were created by inserting a spherical volume into the image and setting the signal to 0 within the volume. The artifacts were all centered over the second molar in the right maxilla and spherical in shape with increasing diameters from 0.5 to 5 cm. The difference between the baseline image, without artifact, and each reconstructed image with an artificial artifact was calculated. The normalized difference between the baseline PET and artifact-corrected PET was used to produce a contour map showing the percentage difference from baseline in each area of the image.

### Statistics

Statistic evaluation was performed with statistical software (SPSS Statistics 22.0, Chicago, IL, USA). The LAVA-Flex sequence was used as a reference. Differences in signal void areas were assessed with the Wilcoxon signed-rank test (Kolmogorov-Smirnov test: *p* < 0.05). Absolute and relative reduction of artifact sizes were calculated for T1-FSE, MAVRIC SL, and MAVRIC-fast sequences and compared using the Wilcoxon signed-rank test. Differences in scores for the qualitative data (anatomic distinction, blurring, or image noise) were compared using the Wilcoxon signed-rank test. Significance level was set at a *p* value of <0.05. Agreement between the two readers was determined using Cohen's kappa, with *κ* values of 0 indicating poor agreement, 0.01 to 0.2 slight agreement, 0.21 to 0.40 fair agreement, 0.41 to 0.60 moderate agreement, 0.61 to 0.80 good agreement, and 0.81 to 1 excellent agreement [31]. Isocontour maps showing the percentage difference between PET scans after AC with baseline MR images and MR images with increasing artifact size were calculated using MATLAB Software version 2013b (MathWorks Inc., Natick, MA, USA).

## Results

A total of 46 dental implants could be identified in the 25 patients with an average age of 60 years (range 28 to 76 years) and average weight of 74 kg (range 44 to 109 kg). Image quality and acquisition were acceptable for all patients.

### Quantitative assessment

The largest artifact size of 612 mm^2^ on axial images was observed on LAVA-Flex sequences and could be reduced to 195 mm^2^ for T1-FSE sequence to 131 mm^2^ for MAVRIC SL and to 126 mm^2^ for MAVRIC-fast (Table 1).

**Table 1:** **Overview of artifact sizes in axial slides from dental alloys in the applied sequences**

	**Minimum**	**Maximum**	**Mean**	**SD**
Size of artifact (mm^2^)				
LAVA-Flex	81.7	2,711.5	611.7	578.2
T1-FSE	36.3	1,169.0	194.9	210.6
MAVRIC	15.2	891.6	130.8	157.3
MAVRIC-fast	11.9	880.6	125.9	167.8
Absolute reduction of artifact (mm^2^), compared to LAVA-Flex				
T1-FSE	−3.7	−2,009.5	−416.8	417.9
MAVRIC	−11.5	−2,287.5	−480.9	463.6
MAVRIC-fast	−39.2	−2,208.8	−485.8	450.3
Relative reduction of artifact (%), compared to LAVA-Flex				
T1-FSE	−04	−88	−62	19
MAVRIC	−11	−96	−75	19
MAVRIC-fast	−37	−96	−78	17

SD standard deviation.

**Figure 1:**
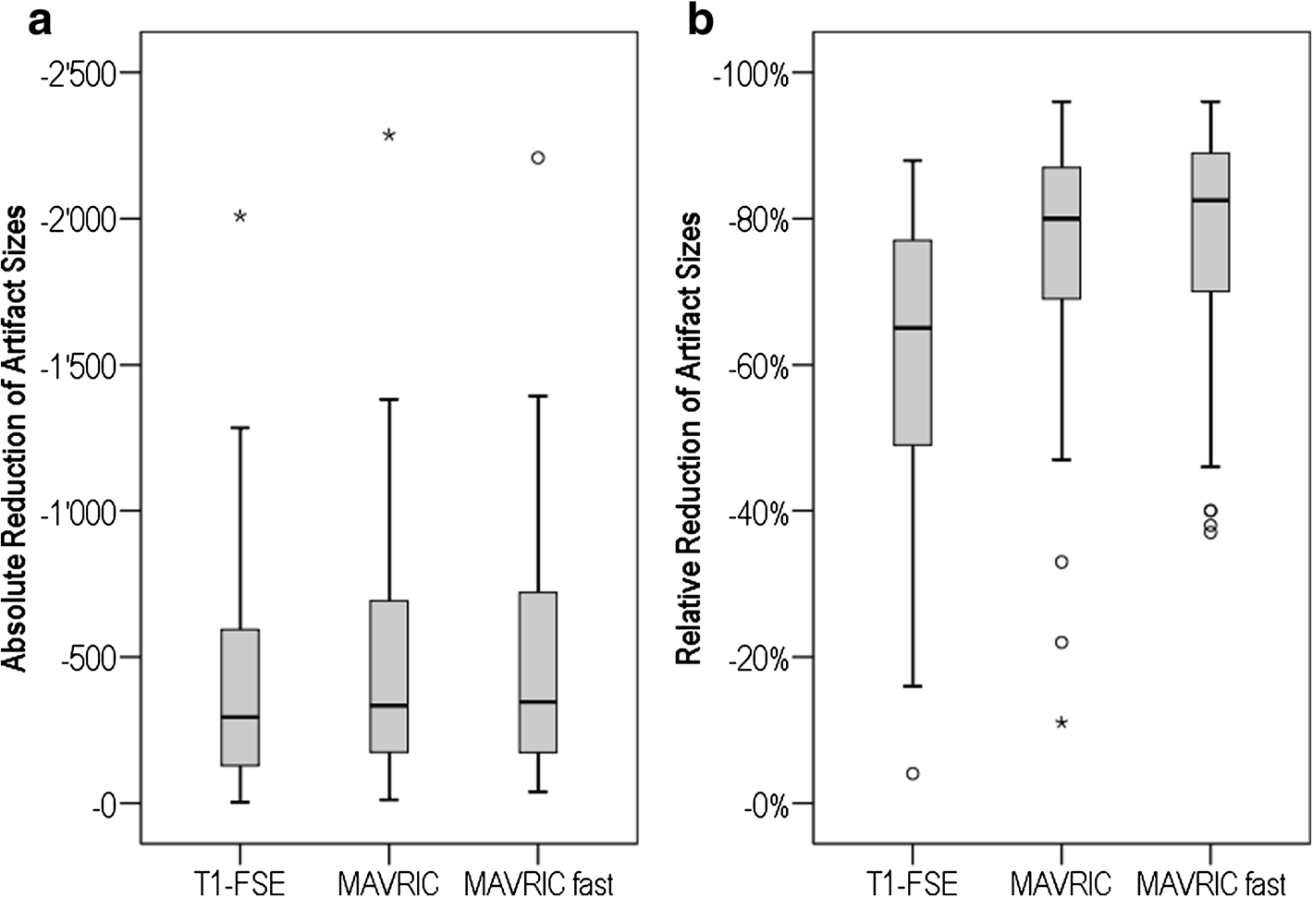
**Box plot illustrating the absolute and relative reduction of artifact size in comparison to LAVA-Flex. (a)** Box plot illustrating the absolute reduction of artifact size in comparison to LAVA-Flex (mm^2^). All three sequences show a broad spread of reduction, reaching from 4 up to 2,010 mm^2^ for T1-FSE, 12 up to 2,288 mm^2^ for MAVRIC, and from 39 up to 2,209 mm^2^ for MAVRIC-fast (Table 1). **(b)** Box plot illustrating the relative reduction of artifact size in comparison to LAVA-Flex (%). The relative artifact reduction was significantly higher for MAVRIC sequences compared to T1-FSE, with a mean of −62% for the T1-FSE sequence, −75% for MAVRIC, and −78% for MAVRIC-fast (Table 1).

Using LAVA-Flex as a reference, the absolute artifact reduction for T1-FSE was smaller (mean 417 mm^2^) than that for MAVRIC SL with a mean of 481 mm^2^ or MAVRIC-fast with a mean of 486 mm^2^ (*p* < 0.001). There was no statistically significant difference between the absolute reduction of MAVRIC SL and MAVRIC-fast (*p* = 0.064) (Table 1, Figure 1a).

The relative artifact reduction with T1-FSE showed a mean of −62%. Both MAVRIC SL and MAVRIC-fast improved the artifact reduction compared to T1-FSE with a mean of −75% (*p* < 0.001) and −78% (*p* < 0.001), respectively (Table 1, Figure 1b). MAVRIC-fast improved relative artifact reduction compared to MAVRIC SL significantly (*p* = 0.017).

### Qualitative assessment

**Figure 2:**
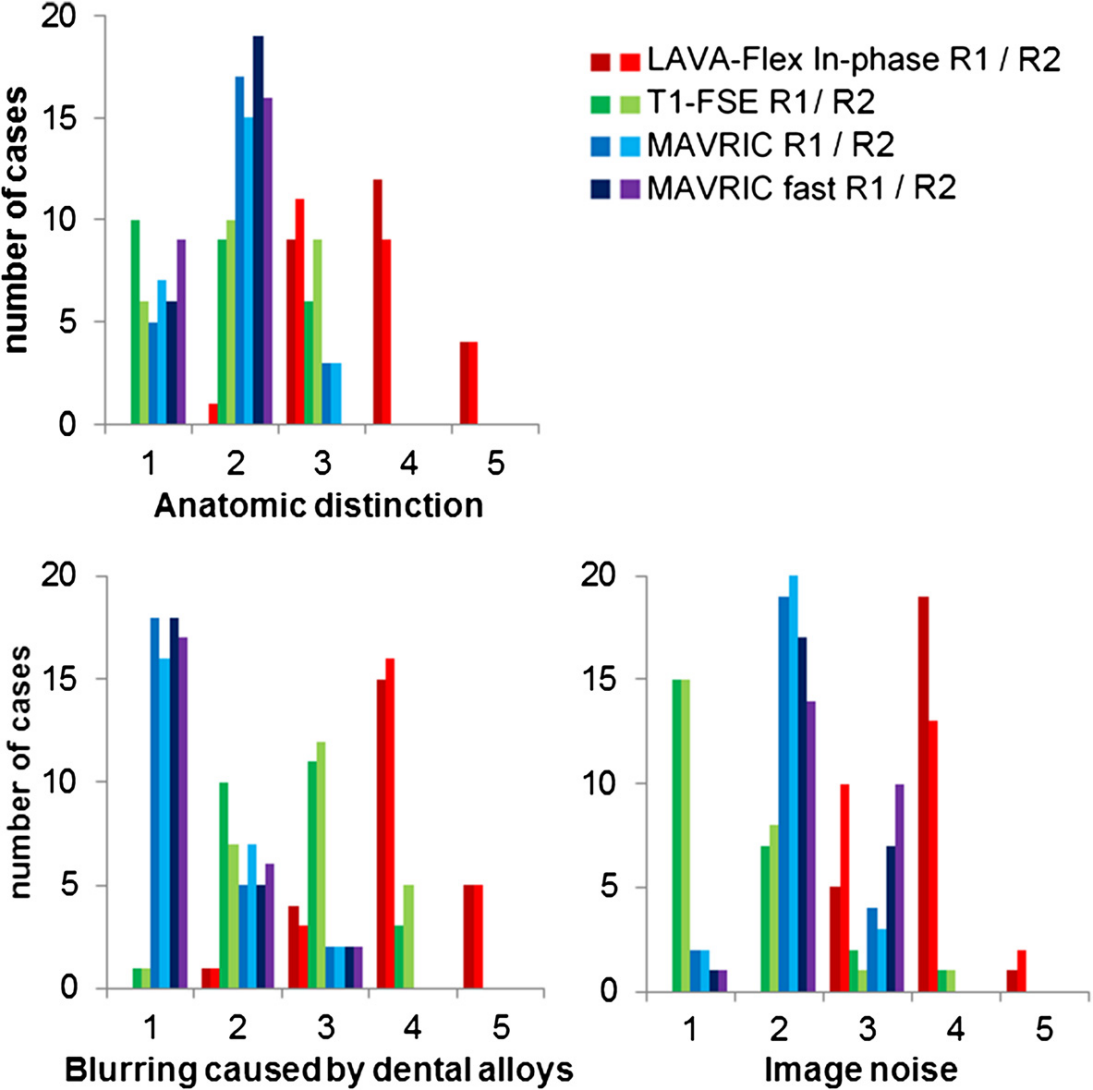
**Qualitative analysis of all four sequences for each patient (** ***n*** **= 25) by two readers (R1/R2).** For anatomic distinction of the floor of the mouth (with 1 = good depiction of anatomical structures, 2 = structures visible with slight blurring, 3 = oral cavity visible with substantial blurring, 4 = oral cavity only partially visible, and 5 = oral cavity not assessable) as well as blurring caused by dental alloys and image noise assessed on a five-point scale (1 = no artifacts, 2 = barely visible artifacts, 3 = visible artifacts without diagnostic impairment, 4 = diagnostic impairment, and 5 = severe artifacts, non-diagnostic).

The worst image quality for all three criteria was noted for LAVA-Flex images by both readers with a mean of 3.80 (±0.71) and 3.64 (±0.81) for anatomic distinction, 3.96 (±0.74) and 4.00 (±0.71) for blurring, and 3.84 (±0.47) and 3.68 (±0.63) for image noise, for readers 1 and 2, respectively (Table 2, Figure 2).

**Table 2:** **Overview of qualitative image analysis and inter-observer agreement**

	**Reader 1**	**Reader 2**	**Kappa (** ***κ*** **)**	**Agreement**
LAVA-Flex				
Distinction of anatomy	3.8 ± 0.7	3.6 ± 0.8	0.75	Good
Blurring	4.0 ± 0.7	4.0 ± 0.7	0.93	Excellent
Noise	3.8 ± 0.5	3.7 ± 0.6	0.54	Moderate
T1-FSE				
Distinction of anatomy	1.8 ± 0.8	2.1 ± 0.8	0.58	Moderate
Blurring	2.6 ± 0.7	2.8 ± 0.8	0.69	Good
Noise	1.6 ± 0.8	1.5 ± 0.8	0.63	Good
MAVRIC				
Distinction of anatomy	1.9 ± 0.6	1.8 ± 0.6	0.85	Excellent
Blurring	1.4 ± 0.6	1.4 ± 0.6	0.83	Excellent
Noise	2.1 ± 0.5	2.0 ± 0.4	0.89	Excellent
MAVRIC-fast				
Distinction of anatomy	1.8 ± 0.4	1.6 ± 0.5	0.72	Good
Blurring	1.4 ± 0.6	1.4 ± 0.6	0.91	Excellent
Noise	2.2 ± 0.5	2.4 ± 0.6	0.76	Good

Qualitative image assessment was done by two readers using a five-point scale from 1 (good depiction/no artifacts) to 5 (not assessable/non-diagnostic). Data are mean ± standard deviation. Agreement rating: *κ* = 0 no agreement; 0.01 to 0.2 slight agreement; 0.21 to 0.40 fair agreement; 0.41 to 0.60 moderate agreement; 0.61 to 0.80 good agreement; and 0.81 to 1 excellent agreement.

There was an increase in image noise for MAVRIC-fast compared to MAVRIC SL for both readers, reaching statistical significance for reader 2 (*p* = 0.011). For anatomical distinction or spatial blurring, there was no relevant difference between MAVRIC SL and MAVRIC-fast.

Regarding spatial blurring, T1-FSE had substantially more artifacts with a mean of 2.64 (±0.76) and 2.84 (±0.80) than MAVRIC SL (mean 1.36 (±0.64) and 1.44 (±0.65), *p* < 0.001) or MAVRIC-fast (mean 1.36 (±0.63) and 1.40 (±0.65), *p* < 0.001). Anatomical distinction was overall slightly better for MAVRIC-fast compared to T1-FSE. However, both readers rated image noise significantly better for T1-FSE compared to MAVRIC SL and MAVRIC-fast (*p* < 0.001).

**Figure 3:**
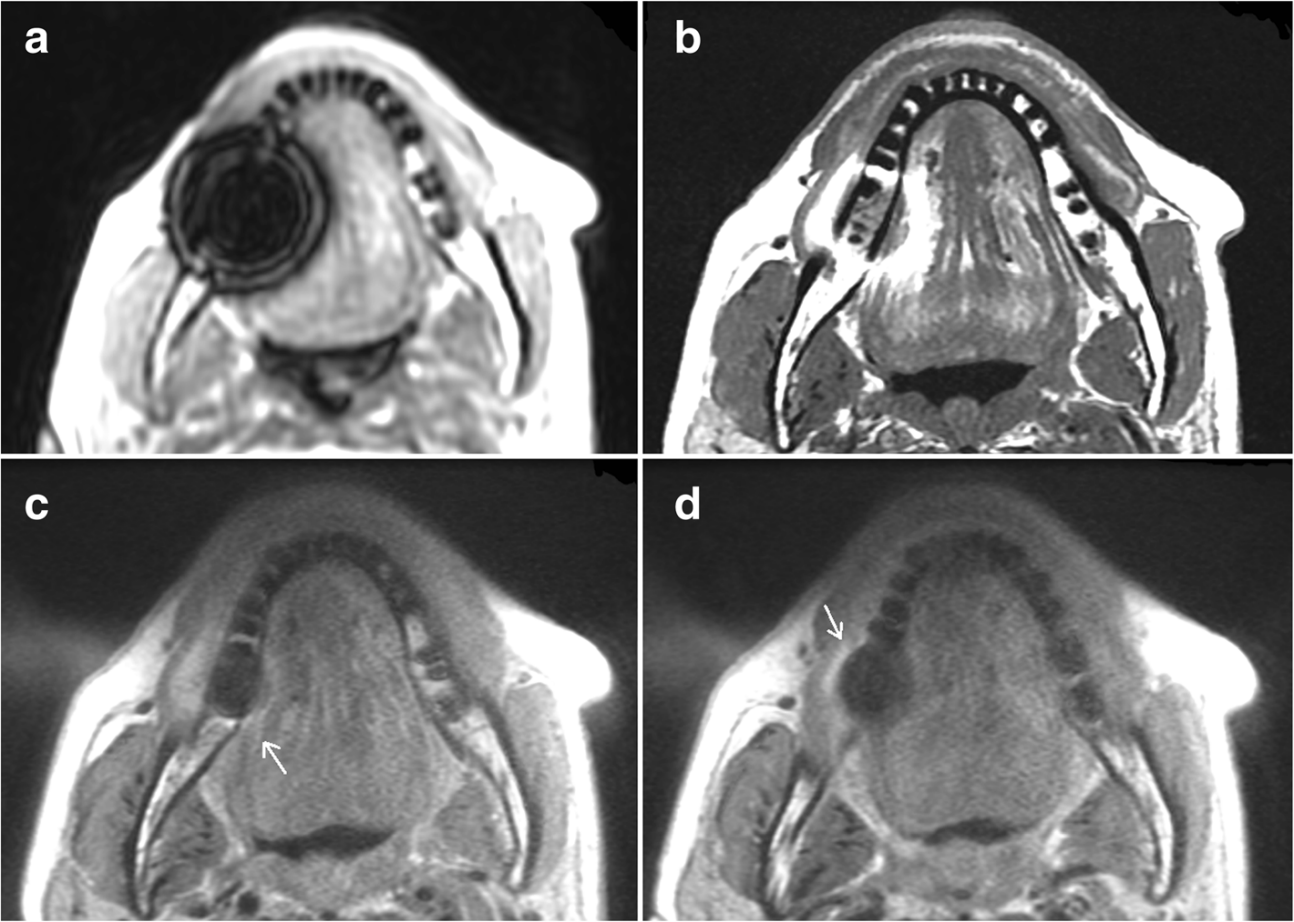
**Different appearances of artifacts.** In the LAVA-Flex images **(a)**, artifacts often showed multiple hyperintense rings in the signal void. On T1-FSE images **(b)** in 12 patients, implants still caused strong hyperintense rings. For MAVRIC **(c)** and MAVRIC-fast **(d)**, hyperintense signals were recorded only in this single case where the dental alloy caused strong artifacts in all sequences.

On 23 (92%) of the images of the LAVA-Flex sequence, the artifacts by the dental alloys showed multiple hyperintense rings (Figure 3). Both readers identified hyperintense ring artifacts on T1-FSE images in 12 cases (48%), while such an artifact was visible only in one case on MAVRIC SL and MAVRIC-fast sequences.

For anatomical distinction, a moderate to excellent inter-observer agreement was noted (*κ* = 0.58 to 0.85). For image quality, inter-observer agreement is good to excellent regarding blurring (*κ* = 0.69 to 0.93) and moderate to excellent for image noise (*κ* = 0.54 to 0.89) (Table 2).

Subgroup analysis for patients with low to moderate (1 to 3) blurring artifacts on LAVA-Flex sequence (group 1) and patients with extensive to non-diagnostic blurring (group 2) was performed. For both readers, there was no significant improvement of spatial blurring with MAVRIC-fast compared to T1-FSE (*p* = 0.102) in group 1. For group 2, MAVRIC-fast led to only barely visible artifacts (mean 1.4 and 1.5, respectively), while T1-FSE showed artifacts impairing diagnostic accuracy in four cases with a mean of 2.8 and 3.0, respectively (Table 3).

**Table 3:** **Image quality of T1-FSE and MAVRIC-fast depending on LAVA-Flex assessment concerning blurring**

	**Reader 1**			**Reader 2**		
	**T1-FSE**	**MAVRIC-fast**	***p*** **value**	**T1-FSE**	**MAVRIC-fast**	***p*** **value**
Distinction of anatomy						
Group 1	1.00 ± 0.00	1.80 ± 0.45	0.046	1.25 ± 0.50	1.50 ± 0.58	0.564
Group 2	2.05 ± 0.76	1.75 ± 0.44	0.109	2.29 ± 0.72	1.67 ± 0.48	0.001*
Blurring						
Group 1	2.00 ± 0.71	1.20 ± 0.45	0.102	2.00 ± 0.82	1.00 ± 0.00	0.102
Group 2	2.80 ± 0.70	1.40 ± 0.68	<0.001*	3.00 ± 0.71	1.48 ± 0.68	<0.001*
Noise						
Group 1	1.00 ± 0.00	2.00 ± 0.71	0.059	1.00 ± 0.00	2.00 ± 0.82	0.102
Group 2	1.70 ± 0.87	2.30 ± 0.47	0.003*	1.62 ± 0.81	2.43 ± 0.51	<0.001*

Image quality was assessed by two readers using a five-point scale from 1 (good depiction/no artifacts) to 5 (not assessable/non-diagnostic), 3 = without diagnostic impairment and 4 = with impairment. Data are mean ± standard deviation. After Bonferroni correction, statistical significance is denoted by *p* < 0.0167 (* = statistically significant). Group 1: blurring by dental alloys in LAVA-Flex was rated 1, 2, or 3. Group 2: blurring by dental alloys in LAVA-Flex was rated 4 or 5.

### Effect of MRI-based PET AC

**Figure 4:**
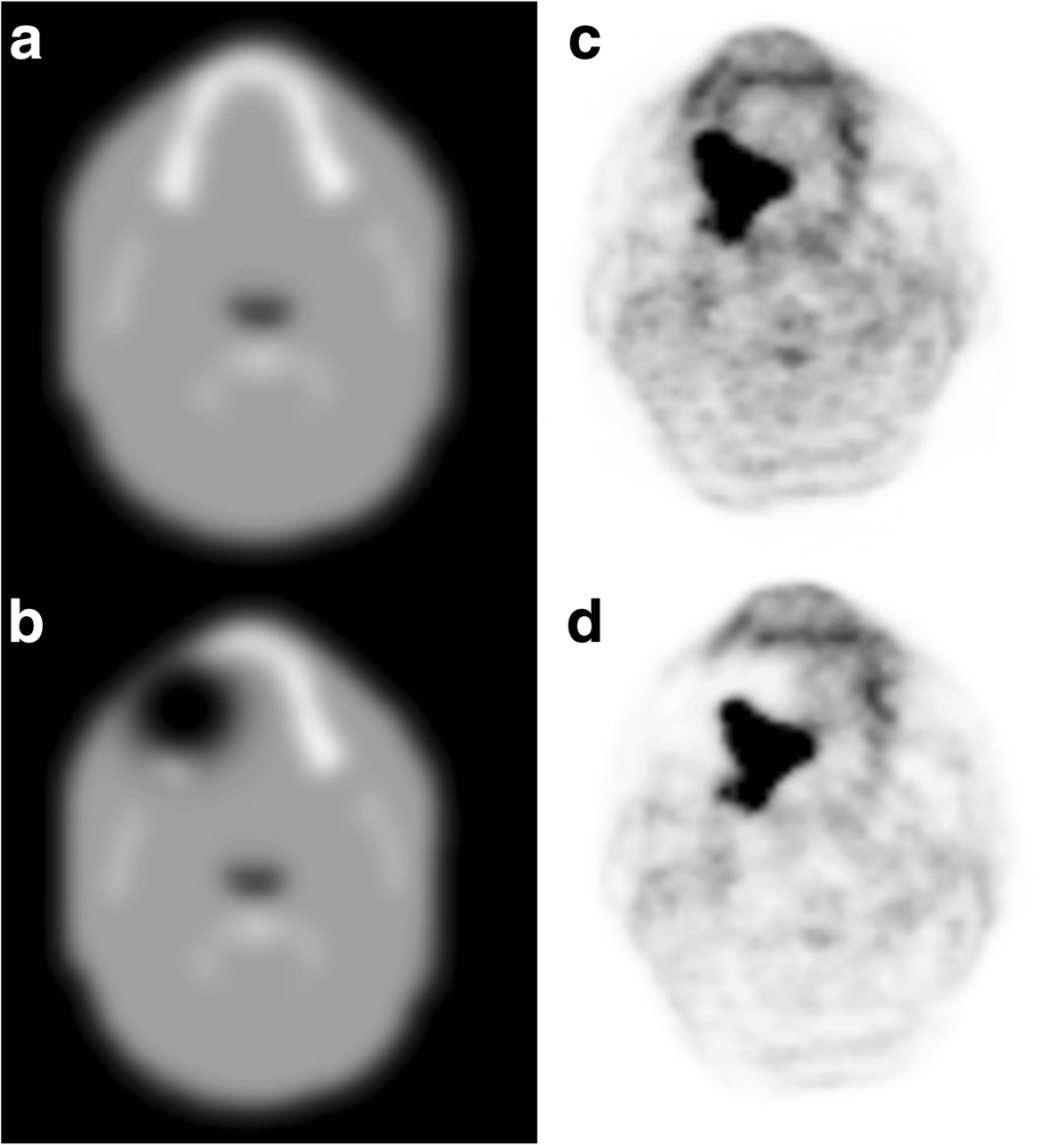
**Axial PET images after attenuation correction without and with artifact. (A)** Axial image of the used MR atlas image for attenuation correction. **(B)** The same image with the inserted artifact over the second left molar. **(C)** Base line axial PET image after attenuation correction using the original MR atlas. **(D)** Corresponding axial PET image after attenuation correction with signal void.

The atlas-based MRI AC of the PET data yielded reference PET values in the tumor with a SUV_max_ of 25 g/ml. The tumor was located at the base of the tongue with a size of 2.3 × 3.7 × 3 cm. The distance between the hottest voxel within the tumor and the center of the artifact was 5 mm. The absolute and relative change of SUV_max_ with increasing diameters of the artificial artifact is given in Table 4. While a signal void of 0.5 cm did not cause any significant change (−0.1%), 5 cm led to substantial underestimation of tumor activity of −33% in our selected case (Figure 4).

**Table 4:** **Change of SUV**_**max**_ **within the tumor with increasing sizes of the artificial artifact**

**Artifact diameter (cm)**	**SUV**_**max**_ **(g/ml)**	**Difference in SUV**_**max**_ **(%)**
0.0	25	0.0
0.5	25	−0.1
1.4	22.9	−8.4
2.3	21.2	−15.1
3.3	20.1	−19.5
4.2	18.2	−27.4
5.2	16.7	−33.4

**Figure 5:**
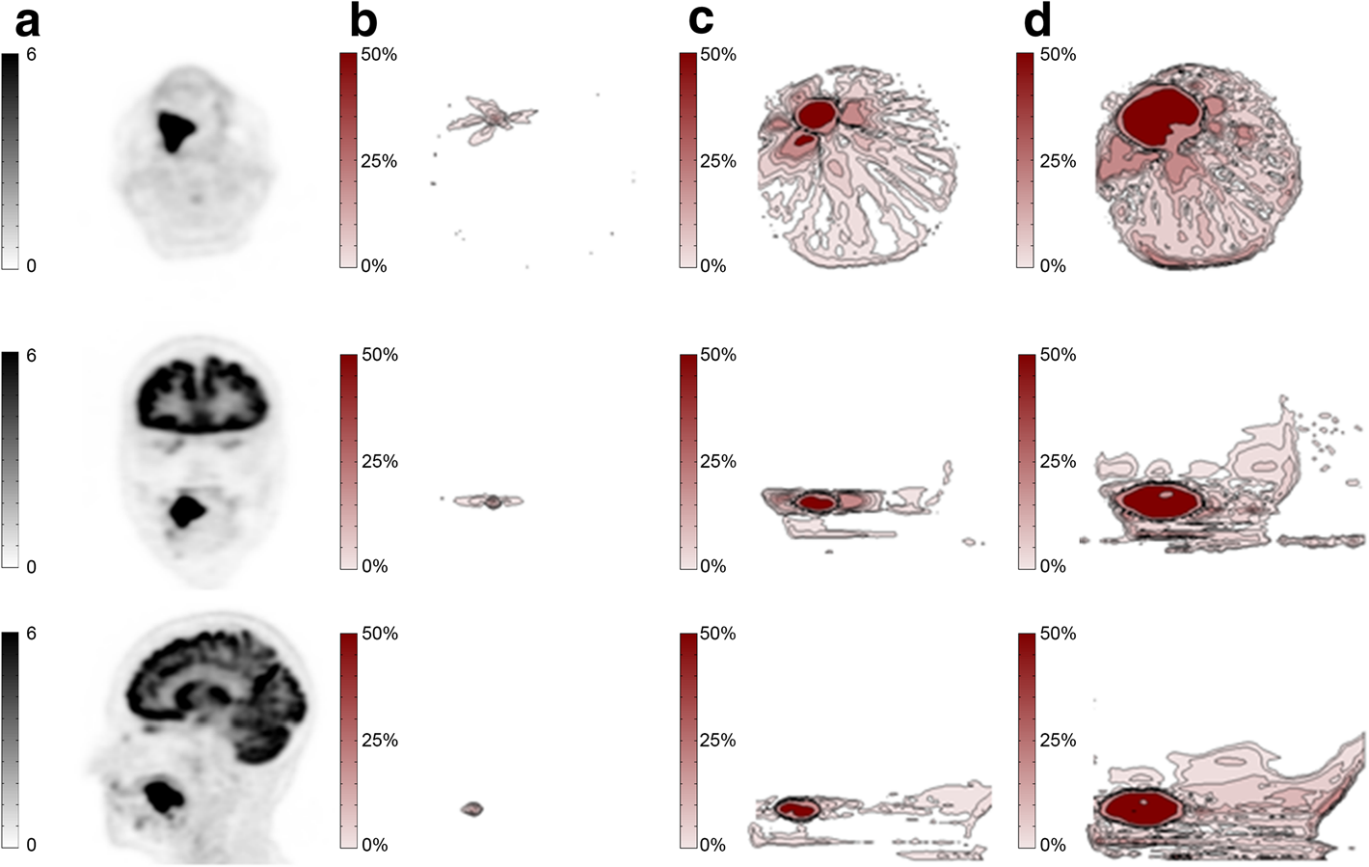
**Different views of the baseline PET image and isocontour plots. (a)** Axial, coronal, and sagittal views of the baseline PET image, corrected with the MR atlas without signal void. **(b-d)** Axial, coronal, and sagittal views of the isocontour plots illustrating the relative difference (from 0% to 50%), between the baseline PET and reconstructed PET images with artificial signal voids with increasing sizes (B = 0.5, C = 2.5, and D = 5 cm).

The isocontour maps illustrating the percentage difference from baseline in each area of the image are given for three cases with increasing artifact sizes from 0.5 to 5 cm (Figure 5).

## Discussion

With this study, we can confirm that MAVRIC is also capable of reducing artifacts from dental implants within the oral cavity. Taking LAVA-Flex images as a reference, the reduction of the artifact size in the axial plane was around −75% for MAVRIC SL (and −78% for MAVRIC-fast). This is also a significant improvement compared to the artifact reduction accomplished by a T1-FSE sequence with large bandwidth (−62%). There was a very broad range of signal void sizes for all sequences, due to the various sizes and compositions of the dental alloys [15]. For patients with moderate artifacts in LAVA-Flex (group 1, grades 1 to 3), T1-FSE yielded good image quality with small artifacts due to dental alloys. For patients with extensive blurring due to dental alloys in LAVA-Flex (group 2, grades 4 and 5), spatial blurring in T1-FSE was significantly higher compared to MAVRIC-fast (*p* < 0.001 for both readers) (Table 3).

MAVRIC-fast was optimized to reduce the acquisition time of the conventional MAVRIC SL sequence protocol for potential integration into a whole-body PET/MRI protocol. By increasing the phase acceleration from 2 to 3, the echo train length (ETL) was reduced. As susceptibility artifacts increase with echo time [32], this time optimization step also resulted in a further reduction of the size of signal voids. On the other hand, the signal-to-noise ratio decreases with phase acceleration leading to an overall higher image noise for MAVRIC-fast compared to MAVRIC (Table 2). An increase of phase acceleration of MAVRIC, therefore, might only be feasible in areas with a sufficient signal-to-noise ratio obtained by a dedicated receiver coil. In the oral cavity, blurring by dental alloys was approximately equal for MAVRIC SL and MAVRIC-fast, but the MAVRIC-fast technique with TR of 3,000 ms resulted in an acquisition time of 3.5 min, compared to 6 min for MAVRIC SL. This gives a reasonable scan time for clinical use, comparable with that of the T1-FSE sequence of 3.2 min. Overall, MAVRIC-fast has shown similar imaging results as MAVRIC SL within a favorable acquisition time.

**Figure 6:**
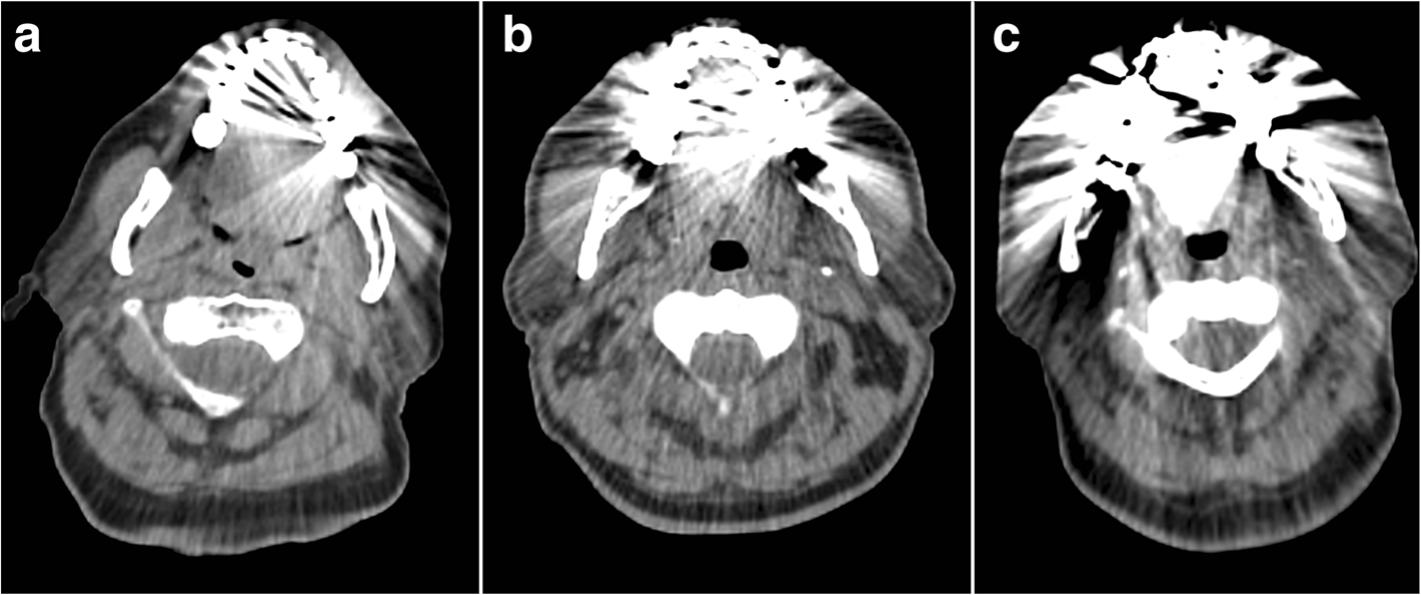
**Artifacts by dental implants on the CT images. (a)** An axial CT image of the same patient as the MRI images in Figure 3. **(b,** **c)** Other patients included in this study.

There was only one patient with hyperintense signals due to dental implants distorting MAVRIC SL/MAVRIC-fast images, while T1-FSE images had this artifact in 12 cases (48%). Such artifacts can lead to non-interpretable images and misdiagnoses; it is therefore favorable to reduce them as much as possible. Furthermore, MRI data are used for AC of PET data in PET/MRI hybrid systems. High image quality without substantial signal voids is favorable for AC [22]. For CT-based AC, it is well known that metal artifacts can lead to false positive findings around prosthesis in PET/CT [33]. In our study, all the obtained CT images showed strong metal artifacts caused by the dental alloys (Figure 6). Therefore, CT-based AC is not a reliable gold standard either. Compared to CT-based AC, the DIXON-based MRI AC (LAVA-Flex) is rather underestimating PET activity in areas of large signal voids [34]. Therefore, large signal voids impair PET AC for PET/MRI [35]*.* The size of signal voids might not translate into identical signal voids on MR attenuation maps; however, a substantial reduction of artifact size will also generate smaller signal voids on MR attenuation maps. The impact of artifact size on PET values could be shown in our simulation study, where artifacts of 19 cm^2^ lead to an underestimation of SUV_max_ of 33%, in a tumor nearby the inserted artifact. In our patient population, artifact size was up to 27 cm^2^ for LAVA-Flex but only 8.9 and 8.8 cm^2^ for MAVRIC SL and MAVRIC-fast, respectively. Nevertheless, the presented MRI sequences could not completely reduce artifacts from dental implants and also will not take into account the true attenuation from metal implants, teeth, or bones.

In addition to a potential improvement of CT-based AC [33], MRI also increases the soft tissue contrast of the oral cavity. This is especially beneficial in FDG PET, since muscle activity can lead to variable FDG uptake in the floor of the mouth [36]. To differentiate this physiological FDG activity from malignancy, it is important that the MRI sequence allows a good anatomical depiction of the oral cavity [17]. T1-FSE images showed better spatial resolution than MAVRIC images in earlier publications [25]. This was also true for the oral cavity in our study with less noise for T1-FSE compared to MAVRIC SL and MAVRIC-fast for most cases. On the other hand, there was a significantly better reduction of spatial blurring for MAVRIC-fast compared to T1-FSE in patients with large artifacts on LAVA Flex (group 2, Table 3). Regarding image fusion, artifact size did not affect the coregistration between MRI and PET in our trimodality setup, since hardware-based coregistration is independent of image information and visual corrections, which if necessary are performed using bony structures, not affected by focal signal voids.

One limitation of this study is that we could not investigate the alloy composition of the dental implants and had no information of their exact material. Therefore, the influence of different materials on the artifacts in the applied sequences could not be addressed. However, this is the most realistic clinical situation, as patients usually are not aware of the exact composition of their dental implants. A further limitation is a certain uncertainty for measurements that might lead to a slight variability in measured sizes of signal voids and that we only compared vendor-specific sequences and could not investigate SEMAC or compare MAVRIC with SEMAC. Also, volume-selective 3D multispectral imaging (VS-3D-MSI), a fusion of SEMAC and MAVRIC technique, has been reported to provide excellent artifact suppression. In dental imaging, SEMAC reduced artifacts from dental alloys composed of high-susceptibility materials of more than 50% compared to conventional SE sequences [37]. It might be valuable to have a direct comparison of these two sequences, which have proven their capability of artifact reduction. With the current study, we could not implement MAVRIC-fast for direct AC correction and therefore could not quantify the direct effect on SUV values. With the additional simulation study, we however could show the potential impact of different artifact sizes on SUV values in a real tumor. Future studies with integration of MAVRIC-fast information into the AC map will be needed to further analyze this aspect.

## Conclusions

In summary, this is the first study showing that MAVRIC-fast could be integrated into whole-body PET/MRI imaging for patients with large signal voids due to dental alloys. MAVRIC SL reduced artifact size and increased precise anatomic delineation of the oral cavity. This reduction should improve the accuracy of PET quantification - since large signal voids lead to substantial SUV underestimation. With MAVRIC-fast, acquisition time was kept reasonably short, slightly worsening image noise but further reducing artifact size compared to T1-FSE or MAVRIC SL. Therefore, MAVRIC-fast could be integrated into the diagnostic imaging work-up for head and neck cancer evaluation in patients with dental alloys and potentially also improve AC.
